# Potential Anionic Substances Binding to Platelet Factor 4 in Vaccine-Induced Thrombotic Thrombocytopenia of ChAdOx1-S Vaccine for SARS-CoV-2

**DOI:** 10.3389/fimmu.2021.782335

**Published:** 2022-01-12

**Authors:** Xiaocong Pang, Haitao Liu, Xu He, Tianrong Ji, Yizhun Zhu, Yimin Cui

**Affiliations:** ^1^Department of Pharmacy, Peking University First Hospital, Beijing, China; ^2^Investment Department, Tigermed Consulting Co., Ltd, Hangzhou, China; ^3^School of Pharmacy and State Key Laboratory for the Quality Research of Chinese Medicine, Macau University of Science and Technology, Macao, Macao SAR, China; ^4^Shanghai Key Laboratory of Bioactive Small Molecules, Department of Pharmacology, School of Pharmacy, Fudan University, Shanghai, China

**Keywords:** vaccine-induced thrombotic thrombocytopenia (VITT), ChAdOx1-S vaccine, SARS-CoV-2, anionic substances, PF4

## Abstract

Recent reports of rare ChAdOx1-S vaccine-related venous thrombosis led to the suspension of its usage in several countries. Vaccine-induced thrombotic thrombocytopenia (VITT) is characterized by thrombocytopenia and thrombosis in association with anti-platelet factor 4 (PF4) antibodies. Herein, we propose five potential anionic substances of the ChAdOx1-S vaccine that can combine with PF4 and trigger VITT, including (1) the proteins on the surface of adenovirus, e.g., negative charged glycoprotein, (2) the adjuvant components of the vaccine, e.g., Tween 80, (3) the DNA of adenovirus, (4) the S protein antigen expressed by the vaccine, and (5) the negatively charged impurity proteins expressed by the vaccine, e.g., adenovirus skeleton proteins. After analysis of each case, we consider the most possible trigger to be the negatively charged impurity proteins expressed by the vaccine. Then, we display the possible extravascular route and intravascular route of the formation of PF4 autoantibodies triggered by the negatively charged impurity proteins, which is accordant with the clinical situation. Accordingly, the susceptible individuals of VITT after ChAdOx1-S vaccination may be people who express negatively charged impurity proteins and reach a certain high titer.

## Introduction

Due to severe thrombotic adverse events named vaccine-induced thrombotic thrombocytopenia (VITT) (1, 2) reported in Denmark, Norway, Germany, Austria, and the United Kingdom, the usage of AstraZeneca recombinant adenoviral ChAdOx1-S was limited in several countries (3). VITT was more frequent in young people, therefore, the health authorities of several European countries and Canada modified their immunization strategies, reserving the ChAdOx1-S vaccine for older people (4). The United States also reported similar events related to the Ad26.COV2-S Janssen vaccine, leading to a pause in its roll-out (4, 5). According to a recent report (6), as of July 2021, 342 patients had died in Taiwan after receiving the ChAdOx1-S vaccine which had been supplied with a total of 1.24 million doses since 15 June; the mortality was as high as 287 parts per million.

Even though patients with VITT had similar mortality after two vaccine doses, the VITT occurrence rate was higher in the ChAdOx1-S vaccine (7, 8). Greinacher et al. reported that people receiving ChAdOx1-S had one or more thrombotic complications beginning 5 to 16 days after vaccination (9). So far, most of the reported cases became symptomatic within 30 days of the first dose of the ChAdOx1-S vaccine, and VITT was more frequent in women and patients aged < 55 years (5, 9). VITT patients often showed laboratory signs of disseminated intravascular coagulation with severe thrombocytopenia (9), and most thrombotic complications occurred at unusual sites, particularly cerebral venous sinus thrombosis (CVT). On the basis of such a situation, healthcare authorities advised vaccine recipients who suffered symptoms such as shortness of breath, chest, abdominal, or extremities pain, severe headache, dizziness, visual disturbances, or other neurologic symptoms within 30 days of ChAdOx1-S vaccination should be urgently investigated for VITT by associated laboratory tests (10, 11).

Then, the serious question is, among the various vaccines approved worldwide, why has the ChAdOx1-S vaccine caused so many VITT cases?

## The Key Player: PF4 and Anionic Substances

The ChAdOx1-S vaccine utilizes chimpanzee adenovirus, which is considered safe, as its vaccine vector is not transmitted in humans, but it seems that this may not be the case.

According to a previous report (1), PF4-heparin antibodies were detected in the blood of patients with severe thrombosis, but these patients did not use heparin. So which component produced a similar effect to heparin after the injection of the ChAdOx1-S vaccine, forming the PF4-component complex, and then led to the formation of the PF4 autoantibody, triggering the thrombosis process just like PF4 immune activation in heparin-induced thrombocytopenia (HIT)?

From the perspective of biochemical properties, McGonagle et al. (12) pointed out that PF4 is easily combined with anionic substances, such as DNA, heparin, etc. Then, which anionic substances of the ChAdOx1-S vaccine may bind to PF4?

## Five Potential Anionic Substances

According to the related reports, we suggest five potential anionic substances of the ChAdOx1-S vaccine that can combine with PF4 as follows:

1. The proteins on the surface of adenovirus, for example, negatively charged glycoprotein
2. The adjuvant components of the vaccine, for example, Tween 80
3. The DNA of adenovirus
4. The S protein antigen expressed by the vaccine
5. The negatively charged impurity proteins expressed by the vaccine, for example, adenovirus skeleton proteins

For substance 1, although part of the adenovirus vaccine can enter the blood after intramuscular injection (13), this reason does not sound plausible, because this could not explain the rarity of the clinical observation of VITT. Moreover, even if some people have been infected with human adenovirus before, there are neutralizing antibodies against human adenovirus, when other adenoviruses enter again, the more possible result is the neutralization of adenovirus, not VITT.

For substance 2, the adjuvant components of the vaccine, such as Tween 80 (14), are also anionic, they may enter the blood and combine with PF4 to cause thrombosis theoretically. However, up to now, no relevant literature has been found to prove that they are related to VITT. More importantly, the adjuvant components are widely used in vaccines or other drugs, this also could not explain the rarity of the clinical observation of VITT (15).

As for substance 1 and substance 2, Gresele et al. (16) pointed out that VITT develops usually at least 1 week after vaccination, it is very unlikely that circulating Ad-vector or vaccine excipients would still be present in the blood, rendering more likely alternative explanations, and in particular an immunological reaction.

For substance 3, McGonagle et al. (12) pointed out that local tissue microtrauma, along with local microbleeding and immune cell activity, will bring adenoviral DNA in contact with PF4, which is then taken up by APCs and memory B cell engagement in the regional lymph nodes, leading to substantially increased PF4 autoantibody production. It is related to failed extravascular tissue tolerance mechanisms which are different from HIT. But how could the DNA enveloped in the Chadox1 adenovirus capsid be released to bind with PF4? Although Kircheis R et al. (17) pointed out that among the 50 billion virus particles in each dose, some may break apart and release their DNA, then the Ad26.COV2.S vaccine would also have released DNA and caused the same incidence and severity of VITT, which does not accord with the current situation (18).

For substance 4, in COVID-19 patients, anti-SARS-CoV-2 IgG-spike glycoprotein immune complexes can activate platelets through FcγRIIa. Kadkhoda et al. (13) figured out that adenoviral vectors leak into the circulation, travel to distant sites, and infect permissive cells. Once infected, copious amounts of soluble spike glycoproteins lead to a relatively high level of SARS-CoV-2 spike “antigenemia”. In a person with a prior SARS-CoV-2 infection and/or with cross-reactive antibodies to common coronaviruses (CoVs), a high enough titer of aberrantly glycosylated antibodies would be induced. But if a person is pre-infected with SARS-CoV-2 and there are SARS-CoV-2 antibodies, the vaccine would be ineffective rather than the cause for VITT. As for the cross-reactive antibodies, there are no direct references supporting its relation to VITT. Greinacher et al. (15) found that antibodies tested against PF4 induced by vaccination do not cross-react with the SARS-CoV-2 spike protein, which indicates that the SARS-CoV-2 spike protein may not be a trigger of VITT.

From another perspective, the above four anionic substances are common in other adenovirus-based vaccines, for example, the Ad26.COV2.S vaccine, if they are plausible, which means that the Ad26.COV2.S vaccine would cause the same incidence and severity of VITT as the ChAdOx1-S vaccine, but that is apparently not the situation (18). Or at least for the ChAdOx1-S vaccine, the above four anionic substances cannot cover all substances that may bind to PF4, triggering VITT.

For substance 5, Almuqrin et al. (19) found that the genome of Chadox1 may express low-level adenovirus skeleton protein, although replication-defective in normal cells, 28 kbp of adenovirus genes is delivered to the cell nucleus alongside the SARS-CoV-2 S glycoprotein gene in some cell lines such as A549 and MRC5, and the expression of adenovirus cytoskeleton protein can then be detected. Besides, McGonagle et al. (12) also pointed out that a role for adenoviral proteins has been suggested as a potential factor in VITT in susceptible individuals. Susceptible individuals may be people who express the negatively charged impurity proteins—adenovirus skeleton protein—and reach a certain high titer. Just as the viral RNA can innate immune-driven immunothrombosis in severe COVID-19 pneumonia, the negatively charged proteins in these adenovirus skeleton proteins may play the same role as viral RNA and form adenovirus skeleton protein-PF4 complexes (12), with this adjuvanticity likely contributing to autoantibody development and ultimately thrombosis in VITT.

## The Possible Routes of the Formation of PF4 Autoantibodies Triggered by Negatively Charged Impurity Proteins—Adenovirus Skeleton Proteins

**Figure 1:**
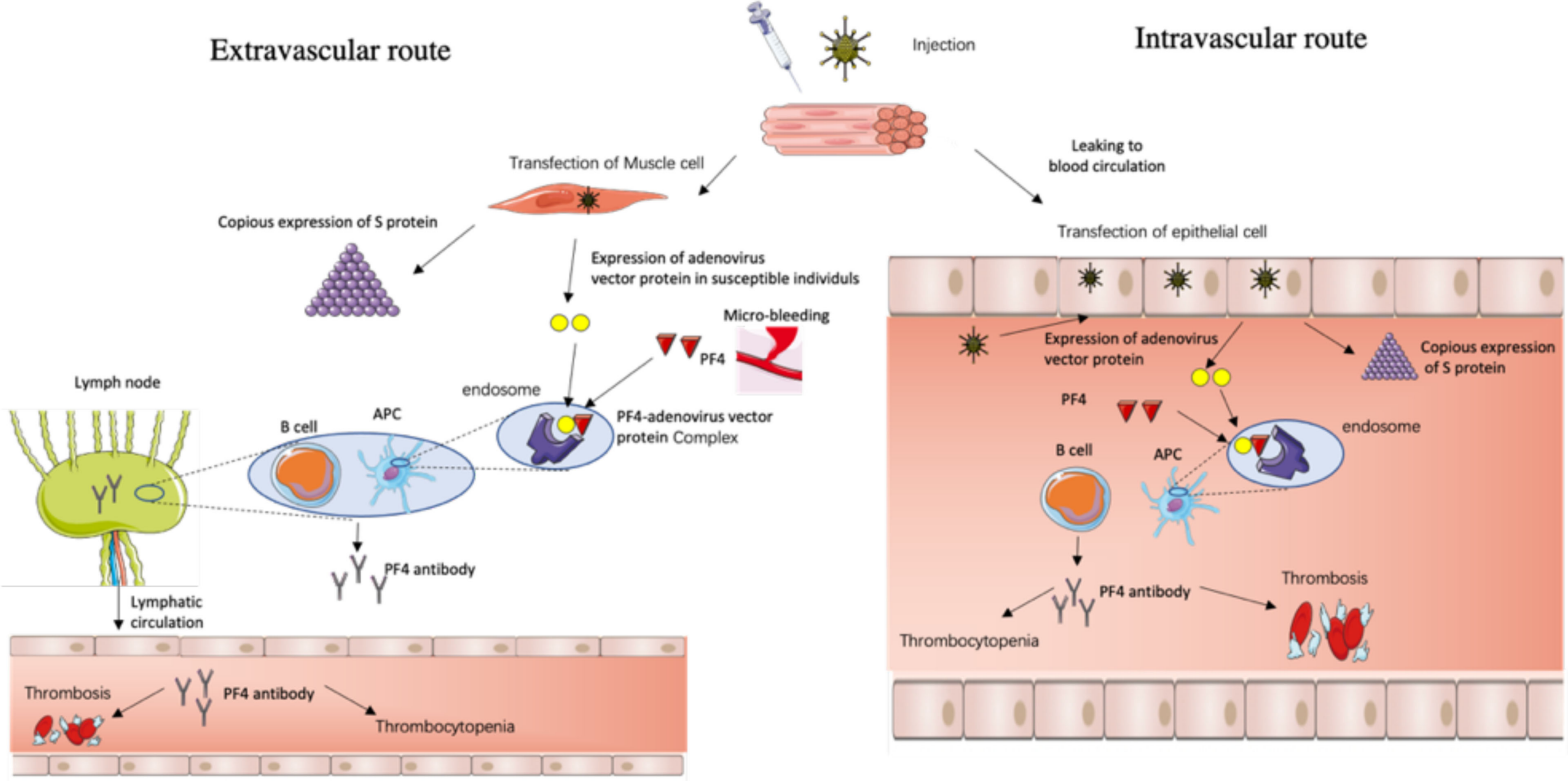
The possible routes for the formation of PF4 autoantibodies triggered by negatively charged impurity proteins—adenovirus skeleton proteins.

The two possible routes for the formation of PF4 autoantibodies include the following:

(1) The extravascular route (**Figure 1**): after the intramuscular injection of the ChAdOx1-S vaccine, the adenovirus enters into the muscle cell, mainly expressing the spike protein, but for some susceptible individuals as depicted above, the adenovirus gene is also expressed and there are adenovirus vector proteins. And if local microbleeding accidently happens, the adenovirus vector proteins have the chance to bind to PF4, which are then taken up by APC cells, memory B cells engage, and regional lymph nodes secret PF4 autoantibody which then enters the blood through lymphatic circulation and triggers the VITT process.

(2) The intravascular route (**Figure 1**): after the intramuscular injection of the ChAdOx1-S vaccine, the adenovirus leaks into the blood circulation, travels to distant and different tissues, and infects a range of permissive cells, which confer susceptibility, such as epithelial and endothelial cells, etc., that then secret the adenovirus vector proteins along with copious SARS-CoV-2 spike proteins, and the adenovirus vector proteins bind to PF4, and trigger the VITT process.

Route 1 is the extravascular immune response hypothesis which takes a relatively short amount of time while route 2 is the intravascular immune response hypothesis which takes a relatively long time, which is concordant with the clinical situation where there are urgent VITT cases occurring about 5 days after vaccination and chronic cases occurring about 24 days after vaccination (1, 12, 18). And young women have a more intense immune response than men and the elderly, so they are more prone to the above immune response routes.

## Discussion

Based on the biochemical properties, we suppose that five possible anionic substances in association with the ChAdOx1-S vaccine may bind to PF4. Although they all have potential, we consider the negatively charged impurity proteins expressed by the vaccine as the most possible trigger, but whether the extravascular and the intravascular models exist together or just one happens needs to be substantiated.

ChAdOx1-S-vaccinated individuals must undergo some concurrent events in order to develop VITT syndrome. (1) They are “predisposed” individuals who could express negatively charged impurity proteins—adenovirus skeleton protein and reach a certain high titer. That “prevalence” of the susceptible individuals determines the basic occurrence rate of VITT, for which the “prevalence” of VITT is different in different countries and regions. (2) For the extravascular route, local accident microbleeding needs happening in unison. (3) For the intravascular route, after the adenoviruses leak into the blood circulation, they need to travel to distant and different tissues, e.g., the cerebral sinus, and infect a range of permissive cells, which would induce lethal cerebral VITT and take a relatively long time in accordance with the cerebral venous thrombosis symptom feature in patients with VITT (20).

We propose one novel and data-supporting (19) potential anionic substance of the ChAdOx1-S vaccine that may combine with PF4 and trigger VITT, which could be verified by testing whether the negatively charged impurity proteins expressed by the vaccine could be detected in the sera from VITT patients through quantitative proteomics, or by separation and purification of the impurity proteins and testing their binding ability with PF4.

Although the mechanism of VITT is still unclear, there are many other hypotheses exploring the potential triggering causes of VITT, such as soluble shorter spiker protein variants binding to PF4, genetically determined enhanced expression of FcγRIIa, and the altered glycosylation state of IgG (16), etc., all of which need hypothetical condition support and verification.

The anionic substances discussed in this article that may bind to PF4 are mainly for the ChAdOx1-S vaccine, other adenovirus-based vaccines need further investigation.

## Data Availability Statement

The original contributions presented in the study are included in the article/supplementary material. Further inquiries can be directed to the corresponding authors.

## Author Contributions

XP, HL, YZ, and YC developed ideas and drafted the manuscript. XH and TJ acquired the data and revised the manuscript. All the authors participated in revising the manuscript and approved the final version. XP and HL contributed equally to this work.

## Funding

This research was funded by the National Key R&D Program of China (grant No. 2020YFC2008304), National Natural Science Foundation (No. 81973320, No.81673509, and No. 81903714) of China and the Macau Science and Technology Development Fund (FDCT) (No. 0002/2019/APD, 067/2018/A2, 033/2017/AMJ, and 0007/2019/AKP).

## Conflict of Interest

HL and TJ are employed by Tigermed Consulting Co., Ltd.

The remaining authors declare that the research was conducted in the absence of any commercial or financial relationships that could be construed as a potential conflict of interest.

## Publisher’s Note

All claims expressed in this article are solely those of the authors and do not necessarily represent those of their affiliated organizations, or those of the publisher, the editors and the reviewers. Any product that may be evaluated in this article, or claim that may be made by its manufacturer, is not guaranteed or endorsed by the publisher.
